# Association of TLR4 Rs4986791 Polymorphism and TLR9 Haplotypes with Acute Myeloid Leukemia Susceptibility: A Case-Control Study of Adult Patients

**DOI:** 10.3390/jpm12030409

**Published:** 2022-03-06

**Authors:** Claudia Banescu, Florin Tripon, Anca S. Bojan, Adrian P. Trifa, Carmen Muntean, George Andrei Crauciuc, Alina Boglis, Marcela Candea, Erzsebet Lazar, Laura Jimbu, Mihaela Iancu

**Affiliations:** 1 Center for Advanced Medical and Pharmaceutical Research, “George Emil Palade” University of Medicine, Pharmacy, Science and Technology, 38, Gheorghe Marinescu Street, 540139 Targu Mures, Romania; tripon.florin.2010@gmail.com (F.T.); andrei.crauciuc@gmail.com (G.A.C.); alinaboglis@gmail.com (A.B.); 2 Genetics Department, George Emil Palade University of Medicine, Pharmacy, Science, and Technology of Targu Mures, 540139 Targu Mures, Romania; 3 Genetics Laboratory, County Emergency Clinical Hospital of Targu Mures, 50, Gheorghe Marinescu Street, 540136 Targu Mures, Romania; 4 Department of Hematology, The Oncology Institute “Ion Chiricuta” Cluj-Napoca, “Iuliu Hatieganu” University of Medicine and Pharmacy, 8, Victor Babes, Street, 400012 Cluj-Napoca, Romania; ancasbojan@yahoo.ca (A.S.B.); jimbulaura@yahoo.com (L.J.); 5 Department of Medical Genetics, “Iuliu Hatieganu” University of Medicine and Pharmacy, 8, Victor Babes, Street, 400012 Cluj-Napoca, Romania; trifa.adrian@gmail.com; 6 Department of Genetics, The Oncology Institute “Ion Chiricuta” Cluj-Napoca, 34–36 Republicii Street, 400015 Cluj-Napoca, Romania; 7 Department of Clinical Science, George Emil Palade University of Medicine, Pharmacy, Science, and Technology of Targu Mures, 540139 Targu Mures, Romania; duicucarmen@yahoo.com; 8 Department of Internal Medicine, George Emil Palade University of Medicine, Pharmacy, Science, and Technology of Targu Mures, 540139 Targu Mures, Romania; marcela1212ro@yahoo.com (M.C.); erzsebetlazarbenedek@gmail.com (E.L.); 9 Department of Medical Informatics and Biostatistics, “Iuliu Hatieganu” University of Medicine and Pharmacy, 8, Victor Babes, Street, 400012 Cluj-Napoca, Romania; miancu@umfcluj.ro

**Keywords:** acute myeloid leukemia, Toll-like receptor gene (*TLR*), susceptibility, single nucleotide polymorphisms

## Abstract

Toll-like receptors (TLRs) have an important role in innate immunity, and single nucleotide polymorphisms (SNPs) of *TLR* genes influence the risk of developing hematological malignancies. We aimed to evaluate the effect of *TLR2* (rs5743708), *TLR4* (rs11536889, rs4986790, rs4986791), *TLR9* (rs187084, rs352140, rs5743836) on AML risk, the relation between investigated SNPs and somatic mutations, clinical features, and the overall survival of adult AML patients. All mentioned SNPs were genotyped in 511 AML cases and 503 healthy controls. *DNMT3A* (R882), *FLT3* (D835, ITD), and *NPM1* mutations’ status were investigated in AML patients. *TLR4* rs4986791 was associated with an increased risk of AML under the dominant model (OR = 1.61, 95% CI: 1.001–2.59). Variant genotypes of the *TLR4* rs4986790 or rs4986791 were associated with the odds of developing AML in the codominant model (OR = 3.14; 95% CI: 1.12–8.84; *p* = 0.032). The *TLR9* rs5743836 variant genotype was associated with the *NPM1* mutation (*p* = 0.002). The investigated SNPs were not associated with the *DNMT3A*, *FLT3* mutations and had no significant contribution to the hazard of death after adjusting for covariates. Our findings suggest that *TLR4* rs4986791 is associated with AML susceptibility. The combined variant genotypes of *TLR4* rs4986790 and rs4986791 increase AML risk, the *TLR9* C-G-A haplotype may represent a promising approach to predict a person’s risk for developing AML.

## 1. Introduction

Acute myeloid leukemia (AML), an aggressive malignant hematologic neoplasm with an incidence that is increasing with age, is characterized by complex pathogenicity that arises as a consequence of the accumulation of genetic abnormalities [1]. AML represents the most common type of acute leukemia among adults and is associated with a short survival in adult AML patients (<20% survive 5 years after diagnostic) and challenging treatment, especially in those cases that are not eligible for intensive chemotherapy or allogeneic hematopoietic stem cell transplantation [2,3,4]. Genetic factors play an important role in the occurrence and development of AML and are used in its risk stratification [3,5,6].

It has been reported that the disruption of the immune system is involved in cancer’s pathogenesis [7]. The innate immune system plays a main role in the body’s defense mechanisms against infections and cancers, including leukemia. Toll-like receptors (TLRs) are expressed by innate immune cells but also by various cancer cells [8]. Different reports considered that TLRs have a dual role or “double-edged sword” effect in cancer, the anti-tumoral effects due to efficient immune responses, and the pro-tumoral effects due to the production of pro-inflammatory cytokines and chemokines, anti-apoptotic molecules, and growth factors that increase tumor cell proliferation, promote invasion and metastasis [8,9]. Previous studies have shown that TLRs are expressed in tumor cells, and are involved in tumorigenesis, tumor growth, and metastasis [10,11,12,13].

TLRs play an important role in triggering both the immune response and the inflammatory process. In different cancers, an altered expression of *TLR* genes had been reported [14]. Altered TLR signaling was linked to hematopoietic dysfunction and malignancy. It was underlined that this signaling is implicated in the pathogenesis of hematological malignancies [15]. The potential role of TLRs in hematologic malignancies has been investigated in several studies [16,17]. In addition, several hematopoietic diseases, including myelodysplastic syndromes (MDS), lymphoproliferative disorders, myeloid neoplasms, have been linked to abnormal TLR signaling [15].

Available evidence suggests that polymorphisms within the *TLR* genes may influence the individual’s capacity to respond properly to TLR ligands (exogenous, endogenous, synthetic analogs, fully synthetic small molecule) and that person may be susceptible to tumor development [11,18].

Only a few studies investigated whether gene polymorphisms of innate immunity influenced the risk of developing hematological malignancies, the results being contradictory. Aref et al. reported that *TLR2* rs5743708 single nucleotide polymorphism (SNP) influences the outcome in AML patients, this SNP is associated with short overall survival (OS) and short disease-free survival [19]. Additionally, the *TLR2* rs4696480 SNP has been associated with a decreased risk of chronic lymphocytic leukemia (CLL) and increased the risk of follicular lymphoma [20]. A meta-analysis that included 55 articles suggested that *TLR4* gene polymorphisms, namely *TLR4* rs4986791 and rs11536889, might represent a genetic risk factor for the development of cancer, whereas *TLR4* rs4986790 was not associated with cancer risk [21]. The *TLR4* rs4986790 SNP was associated with the risk of mucosa-associated lymphoid tissue (MALT) lymphoma and Hodgkin’s lymphoma [20].

A small study that included 62 adult patients with AML from Poland showed that *TLR9* rs5743836 and rs187084 gene polymorphisms were associated with predisposition to the disease [16]. *TLR9* rs5743836, polymorphism may represent a molecular risk factor for B-cell Non-Hodgkin lymphomas (B-NHL) among Egyptian subjects [22]. No significant association between rs2066807 and rs187084 SNPs of the *TLR9* genes and the risk of chronic lymphocytic leukemia (CLL) in the Egyptian population was reported previously [23]. None of the published studies investigated the *TLR2* rs5743708, *TLR4* (rs11536889, rs4986790, rs4986791), and *TLR9* (rs187084, rs352140, rs5743836) variants simultaneously.

We carried out this case-control study to estimate the overall AML risk of the *TLR2* rs5743708, *TLR4* rs11536889, *TLR4* rs4986790, *TLR4* rs4986791, *TLR9* rs187084, *TLR9* rs352140, and *TLR9* rs5743836 polymorphisms in a large group of Caucasian adult AML patients. We further analyzed whether the haplotypes of the mentioned gene polymorphisms influence the AML risk. We evaluated the relation between variant genotypes of the *TLR2* rs5743708, *TLR4* (rs11536889, rs4986790, rs4986791), and *TLR9* (rs187084, rs352140, rs5743836) SNPs with somatic mutations (*FLT3, NPM1, DNMT3A)* and clinical features and overall survival of AML patients.

## 2. Materials and Methods

### 2.1. Subjects Included in the Study

We performed a case-control study that included a total of 1014 Eastern European individuals, living in Central and northeastern regions of Romania. The patient group included 511 adult patients with AML diagnosed at the Hematology Clinics from the County Emergency Clinical Hospital of Targu Mures and the Hematology Department of the Oncology Institute “Prof. Dr. Ion Chiricuta” in Cluj-Napoca, Romania. AML patients included in the study were classified into different subtypes in compliance with the criteria of the World Health Organization (WHO) 2016 [24]. The control group included 503 healthy unrelated adults. The inclusion criteria in this group were no evidence of cancer and residence from the same region as the patients.

Approval of the Ethics Committee of the George Emil Palade University of Medicine, Pharmacy, Science and Technology of Targu Mures, Romania has been requested and obtained (no. 1383/2021). The confidentiality of all subjects included in the study was ensured to be in line with the Declaration of Helsinki.

### 2.2. Molecular Analysis

Genomic DNA (gDNA) was extracted from peripheral blood specimens collected in 2 mL EDTA vacutainer tubes by using PureLink Genomic DNA Mini Kits (Thermo Fisher Scientific, Waltham, MA, USA) and Wizard Genomic DNA Purification kits (Promega, Madison, WI, USA) with minor modification [25]. The concentration and purity of the obtained DNA samples were measured by a BioSpectrometer basic (Eppendorf, Wien, Austria).

Genotyping was conducted using a Real-Time PCR platform, namely 7500 Fast Dx Real-Time PCR from Applied Biosystems (Thermo Fisher Scientific, Waltham, MA, USA) and corresponding Applied Biosystems TaqMan SNP Genotyping Assay detecting *TLR2* rs5743708 (ID C__27860663_10), *TLR4* rs11536889 (ID C__31784034_10), *TLR4* rs4986790 (ID C_11722238_20), *TLR4* rs4986791 (ID C_11722237_20), *TLR9* rs187084 (ID C___2301952_10), *TLR9* rs352140 (ID C_2301954_20), and *TLR9* rs5743836 (ID C__32645383_10). Wild-type (mentioned as reference) and variant alleles of the investigated TLR SNPs were specified according to the Ensembl genome browser (ensembl.org).

*DNMT3A* (R882), *FLT3* (D835, ITD), and *NPM1* mutations’ status were investigated in each patient by PCR, restriction fragment length polymorphism PCR (RFLP-PCR), fragment analysis, and capillary sequencing for confirmation, as previously reported [26]. To control the quality of genotyping, about 5% of DNA samples of AML cases and controls were randomly included and distributed in working plates as duplicates and genotypes, and the obtained genotypes were identical.

### 2.3. Statistical Analysis

Quantitative continuous variables with Gaussian distribution were summarized by centrality and dispersion descriptive statistics (mean and standard deviation) while qualitative variables were described using relative and absolute frequencies (%).

The mean comparisons of demographic variables in controls and AML groups were performed using the Student-t test for independent samples. The associations between qualitative characteristics were tested using Chi-square or Fisher’s exact tests.

Binomial logistic regression was used to assess and quantify the association of genotypes, alleles, and combined genotypes of studied SNPs with odds of AML. The association analysis based on unconditional logistic regression was conducted in order to find the odds ratios (OR) with a 95% confidence interval (95% CI) adjusted for age and gender under different genetic models (codominant, dominant, recessive, and allelic).

Hardy–Weinberg equilibrium for each SNP and Linkage disequilibrium (LD) analysis between each pair of SNPs were performed using the “SNPassoc” and “genetics” R package [27,28]. LD pattern was based on the calculation of the unstandardized LD coefficient (D), standardized LD coefficient (D’), Pearson correlation coefficient (r), and Chi-square’s test for significance of LD. The haplotype frequencies and the potential association of estimated haplotypes with the risk of AML were tested using “haplo.stats” R package [29].

Median survival time (MeST) was calculated to estimate the time point when 50% of studied AML patients died. Log-rank test was used to compare the distribution of overall survival time across studied SNPs under the dominant genetic model. The univariable and multivariable Cox proportional-hazards models were used in order to evaluate the genetic SNPs effects on risk of death expressed as hazard ratio (HR) with 95% CIs adjusted for known clinical covariates [age group (≥60 years vs. <60 years), sex (male vs. female), type of AML (WHO classification), ELN 2017 risk groups, ECOG, Eastern Cooperative Oncologic Group status; chemotherapy dose (high dose, low dose); LDH, lactate dehydrogenase (≥600 IU/L vs. 600IU/L); PLT, platelet (>40,000 vs. ≤40,000 cells/mm^3^); WBC, white blood cell (≥50,000 cells/mm^3^ vs. <50,000 cells/mm^3^), hemoglobin level (<10 g/dL vs. ≥10 g/dL), hematocrit level (<36% vs. ≥36%), blasts (in bone marrow, ≥70% vs. <70%), somatic mutations (in *FLT3*, *NPM1*, *DNMT3A* genes)].

The two-sided *p*-value < α = 0.05 was regarded as a statistically significant value in inferential analysis.

All statistical analysis was performed in R v.4.1.1 [30].

## 3. Results

### 3.1. Demographic and Clinical Characteristics of AML Patients and Controls

The mean age of the AML group was 57.22 ± 15.22 years (median 60.0 years, range values: 19–90 years), while of the control group it was 58.56 ± 16.36 years (median 63.0 years, range values: 20–89 years). There was no significant difference between AML and control groups regarding age distribution (*p* = 0.156) and gender frequency (*p* = 0.264). The AML group consisted of 231 females (45.2%) and 280 males (54.8%), whereas the control group included 245 (48.7%) females and 258 (51.3%) males.

White blood cells (WBC) greater than 50,000 cells/mm^3^ were observed in 113 cases (22.1%) while in the 398 of the remaining cases the WBC count was ≤50,000 cells/mm^3^. High lactate dehydrogenase (LDH) level, defined as ≥600 IU/L was noticed in 299 (58.5%) of investigated AML patients. Most of the AML patients (n = 352, 68.9%) had the hemoglobin (Hgb) level lower than 10 g/dL, whereas about one-third (159 cases, 31.1%) had the Hgb level ≥10 g/dL.

Regarding the Eastern Cooperative Oncology Group (ECOG) Performance Status, 202 (39.5%) patients had an ECOG grade of 3, 130 cases (25.4%) had an ECOG grade of 2, 94 (18.4%) had an ECOG grade of 4. A high blast percentage (defined as ≥70%) was found in 194 (38%) AML patients. According to the European Leukemia Net (ELN) 2017 risk stratification [31], 191 (37.38%) of our patients were included in the low-risk group, 204 (39.92%) in the intermediate, 116 (22.70%) in the high-risk (adverse) group.

### 3.2. Genotypic and Allelic Frequencies of TLR2 rs5743708, TLR4 (rs11536889, rs4986790, rs4986791) and TLR9 (rs187084, rs352140, rs5743836) SNPs and Their Effects on AML Susceptibility

The genotypic and allelic distributions of the studied *TLR2*, *TLR4*, *TLR9* gene polymorphisms in AML patients and controls are summarized in Table 1. All TLR genotype frequencies of AML cases satisfied the principles of the Hardy–Weinberg equilibrium (HWE) (*p* > 0.05). Additionally, the genotypes’ distribution of *TLR* SNPs in healthy subjects was in accordance with the HWE (*p* > 0.05).

Also, the genotypes distribution of *TLR* SNPs in healthy subjects was in accordance with the HWE (*p* > 0.05). The *TLR4* (rs11536889, rs4986790, rs4986791) and *TLR9* (rs187084, rs352140, rs5743836) SNPs were in a moderate to strong linkage in AML cases and control groups (*p* < 0.001) (Table 2 and Table 3).

The results of logistic regressions showed that only the *TLR4* rs4986791 SNP was significantly associated with an increased risk of AML under the dominant model (OR = 1.61, 95% CI: [1.001, 2.59]).

No significant difference in the genotype distribution of the other six investigated polymorphisms was found between AML cases and healthy subjects in our large adult population group in any of the inheritance genetic models tested (Table 1).

### 3.3. Haplotype Analysis

We further performed the haplotype analysis which generated eight haplotypes for TLR9 SNPs and six haplotypes in the *TLR4* gene. The C-A-G, C-G-A, T-A-A, and T-G-G haplotypes in the *TLR9* gene polymorphism were associated with increased odds of AML (adjusted OR [95% CI]:1.87 [1.10, 3.19], 5.54 [1.16, 26.51], 2.01 [1.24, 3.28], 3.31 [1.06, 10.34], respectively) when the T-A-G haplotype was the considered reference (Table 4).

The distribution of estimated haplotypes of the *TLR4* (rs11536889, rs4986790, rs4986791) SNPs did not show any significant difference between the AML and control groups.

### 3.4. Association Analysis between Different Combinations of TLR2, TLR4, and TLR9 SNPs and AML Susceptibility

We combined all studied SNPs by the number of variant genotypes (defined based on the dominant model, except for *TLR2* rs5743708) to test their possible joint effect on the odds of AML. The combined variant genotypes were statistically associated with increased odds of AML. We found that the number of variant genotypes varied between 0 (92 subjects with wild-type homozygous genotypes for all SNPs, 9.07%) and 5 [17, 1.678%]. Further, we transformed the number of variant genotypes into a categorical variable with the following categories: 0–1, 2–3, and 4–5 variant genotypes with frequencies of 168 (16.57%), 748 (73.77%), and 98 (9.66%), respectively. Although we noticed an increased odds of AML for subjects carrying 4–5 variant genotypes, the result did not reach statistical significance (OR = 1.11, 95% CI: 0.54–1.05, *p* = 0.099 for 4–5 variant genotypes vs. 0–1 variant genotypes, OR = 0.75, 95% CI: 0.67–1.85, *p* = 0.678 for 2–3 variant genotypes vs. 0–1 variant genotypes).

We also analyzed the joint effect of double combinations of SNPs variant genotypes and we found no significant effect, except for the genotype combination of *TLR4* rs4986790 AG + GG and *TLR4* rs4986791 CT + TT variants (Table 5). The presence of the variant genotypes of the *TLR4* rs4986790 or *TLR4* rs4986791 SNPs in the dominant model was positively associated with the odds of developing AML (OR = 3.14; 95% CI: 1.12–8.84; *p* = 0.032). For both the *TLR4* rs4986790 and *TLR4* rs4986791 gene polymorphisms, we combined both the heterozygous and variant homozygous genotypes as a single group and compared it with the double wild-type genotype. Subjects carrying combined heterozygous and homozygous variant genotype of the two *TLR4* SNPs (rs4986790 and rs4986791) had higher odds of AML compared with individuals possessing double wild-type genotypes (OR = 1.66; 95% CI: 1.05–2.64; *p* = 0.028). We did not find a significant association between the other double combinations of the *TLR2*, *TLR4* and *TLR9* variant genotypes and the odds of AML (*p* > 0.05).

Moreover, we performed all *TLR* SNP–SNP interactions using a log-likelihood ratio test under the dominant genetic model, but we did not find a statistically significant interaction effect of any two SNPs.

The results indicated that the *TLR4* rs4986790 SNP did not have a modifying effect on the odds of AML initiated by the *TLR4* rs4986791 SNP (dominant model, *TLR4* rs4986790 × *TLR4* rs4986791 interaction *p*-value = 0.128). Taking into account the fact that the presence of the combined variant genotype of *TLR4* rs4986790 and *TLR4* rs4986791 was significantly associated with increased odds of AML, we found that these two gene polymorphisms may have a combined effect and no modifying effect on AML susceptibility.

### 3.5. Relationship between Variant Genotype Distribution of TLR2 rs5743708, TLR4 (rs11536889, rs4986790, rs4986791) and TLR9 (rs187084, rs352140, rs5743836) and Somatic Mutations in AML Patients

We further analyzed the relationship between the studied *TLR2*, *TLR4* and *TLR9* SNPs and somatic mutations in AML patients (Supplementary Materials Table S1: Association analysis between variant genotypes/alleles of investigated *TLR* SNPs and odds of AML). The results showed that the *TLR9* rs5743836 variant genotype (AG + GG) was significantly negatively associated with NPM1 mutation (Chi-square test, *p* = 0.002), a higher relative frequency of the *TLR9* rs5743836 variant genotype (AG + GG) being observed in the subgroup without the NPM1 (30.5%) vs. patients with NPM1 mutation (14.4%). We noticed a trend towards statistical significance for the association between the *FLT3* ITD mutations and *TLR4* rs11536889 SNP variant genotype (GC + CC) (Chi-square test, *p* = 0.055), the variant genotype (GC + CC) being observed in 26.6% of the patients without the *FLT3* IDT mutation vs. 37% of the patients with the *FLT3* IDT mutation. No significant associations were found between the *DNMT3A* and *FLT3* mutations and the other investigated SNPs.

### 3.6. Associations between TLR2 rs5743708, TLR4 (rs11536889, rs4986790, rs4986791) and TLR9 (rs187084, rs352140, rs5743836) SNPs and Overall Survival

As shown in Table 6, Kaplan–Meier analysis showed that carriers of the *TLR2* rs5743708 GA genotype had a better overall survival than carriers of the wild genotype (*p* = 0.012). In the separate univariable Cox proportional-hazards models, we observed that only the *TLR2* rs5743708 GA genotype was significantly associated with a decreased HR for death (GA vs. GG: unadjusted HR = 0.54, 95% CI: 0.33-0.88, unadjusted *p* = 0.0137), while a tendency toward statistical significance was noticed for the *TLR4* rs4986790 AG + GG variant genotype (*p* = 0.092) and *TLR4* rs4986791 CT genotype (*p* = 0.070). TLR2 rs5743708 did not remain as an independent predictor for the hazard of death (adjusted *p* = 0.272) after adjusting for other clinical known covariates (Table 6).

## 4. Discussion

A difficulty in understanding the true role of *TLR* SNPs in increased susceptibility to cancer, including the hematological malignancies, has been the limited size of the investigated groups of patients for genetic-associations studies. Furthermore, the lack of replication of positive findings among different studies and the heterogeneity among individual studies did not allow the drawing of firm conclusions regarding the contribution of *TLR* SNPs to cancer pathogenesis [19,21,32,33,34,35,36].

In this study, we sought to address the question of whether the presence of the polymorphisms of the genes involved in the innate immune system, namely *TLR2*, *TLR4* and *TLR9*, correlates with an increased risk for predisposing to AML, considering that there are scarce studies that investigated *TLR2*, *TLR4* and *TLR9* SNPs and the risk of hematological malignancy. The frequency of *TLR2* rs5743708, *TLR4* (rs11536889, rs4986790, rs4986791) variant genotypes is consistent with that reported by Schnetzke et al. in AML patients from Germany [37], but different from that reported by Chen et al. in the Chinese Han population [38]. One explanation for the different frequencies of the genotypes observed in our study and that of Schnetzke et al. (European subjects) and in the study of Chen (Asian subjects) is the ethnic origin. The frequency of the variant allele for investigated SNPs is similar to that reported for 1000 Genomes Project Phase 3.

Moreover, our study investigated the associations between the prognostic indicators in AML and seven SNPs of *TLR* genes, and aimed to figure out the correlation between these SNPs and the known prognostic factors in AML (patient age, mutations in *FLT3*, *NPM1,* and *DNMT3A* genes at diagnosis).

The present study showed that variant genotypes of the *TLR2* rs5743708, *TLR4* (rs11536889, rs4986790), and *TLR9* (rs187084, rs352140, rs5743836) gene polymorphisms were not associated with AML risk. Our findings are partly similar to those reported by other studies previously [14]. Thus, no significant difference between cancers and controls for *TLR2* rs5743708 SNP was reported in a meta-analysis conducted by Wang et al. [32].

Aref et al. included in their study 120 AML patients and 100 healthy subjects from Egypt and reported no significant differences in genotype or alleles frequency between patients and healthy controls regarding *TLR2* rs5743708, *TLR4* rs4986790, and rs4986791 polymorphisms [19].

Recently, a significant association of variant alleles of the *TLR4* rs4986790 and rs4986791 with the risk of development of colorectal cancer was reported by a study that included 268 subjects (127 patients and 141 healthy Egyptians) [33]. Similarly, in the meta-analysis performed by Ding L et al., it has been suggested that polymorphisms, namely *TLR4* rs4986791 and rs11536889, might represent a genetic risk factor for cancer development [21]. In agreement with these findings, our study also indicated that only *TLR4* rs4986791 SNP is associated with an increased risk of AML. Our finding is in contradiction with that reported by Aref et al. [19]. One explanation might be the number of the subjects included in our study (more than 1000), and the ethnic differences between the investigated populations (northeastern Africans and Caucasians).

Moreover, we observed that the presence of the double combination of the variant genotype of the *TLR4* rs4986790 or *TLR4* rs4986791 SNPs in the dominant model was positively associated with the odds of developing AML in investigated Caucasian patients.

The variant genotypes of *TLR4* rs1156889 and *TLR9* rs187084 and rs1927911 SNPs were found to be associated with the increased risk of developing cervical cancer among Indian women [34]. Despite the previous reports, we failed in proving any significant association between the variant genotype of the *TLR9* rs187084 SNP and AML in our cohort of adult patients.

Rybka et al. found that the *TLR9* rs187084 polymorphism was associated with the progression of AML when they investigated a small group of 62 Polish AML patients [16]. In addition, they noticed that *TLR9* rs187084 influenced patients’ survival [16]. This is in contradiction with our study that revealed no associations with the hazard of death in AML cases. An explanation for different results may be represented by the small study group of AML cases and controls investigated by Rybka et al. A previous study suggested that *TLR9* rs187084 influenced the outcome of transplants in AML cases after a myeloablative, non-T-cell-depleted transplant. Furthermore, a reduced relapse rate was observed after transplant in AML patients with the *TLR9* gene variant [35]. Wan GX et al. reported a significantly increased risk of cancer for the *TLR9* rs187084 SNP, especially with cervical cancer, when they stratified analyses by specific tumor types [36].

The variant heterozygous genotype of the *TLR9* rs5743836 SNP conferred almost a fourfold increased risk of B-cell Non-Hodgkin lymphomas (B-NHL) in patients from Egypt (OR = 3.93, 95% CI = 2.16–7.14) [22]. This observation was supported neither by our study nor by the meta-analysis that included 25,685 subjects that suggested that the *TLR9* rs352140 and rs5743836 SNPs were associated with a decreased risk of development of breast and digestive system cancers [34].

Nazarova et al. suggested a relationship between *TLR2* and *TLR3* SNPs with the development of chronic lymphoproliferative diseases and considered that these SNPs might represent early additional diagnostic and prognostic criteria [39]. However, we failed to replicate those findings.

No previous study investigated the impact of haplotypes of the mentioned gene polymorphisms on the AML risk. Our findings revealed that the C-G-A haplotype of *TLR9* was associated with a fivefold increase in AML risk, while C-A-G and T-A-A haplotypes showed an about twofold elevated risk of AML among adult patients.

Ashton et al. investigated the influence of the *TLR9* rs5743836 and rs187084 SNPs haplotype on cancer risk and reported a protective effect of the *TLR9* SNPs haplotype for endometrial cancer risk (OR = 0.11, 95% CI: [0.03–0.44]; *p* = 0.002) [40].

In our study, the haplotypes of the *TLR4* (rs11536889, rs4986790, rs4986791) SNPs showed no association with increased susceptibility to AML. Contrary to our observation, the haplotype analysis of *TLR4* SNPs, (namely AT and GT haplotypes) were associated with increased odds for colorectal cancer (OR = 3.54 and 3.45; 95% CI: 1.48–8.48 and 1.09–10.83, respectively) [33]. The discordant results might be due to the different sample sizes, or to the fact that the effect of the *TLRs*’ haplotype is restricted to certain subgroups of patients.

The individual contribution of the *TLR2*, *TLR4* and *TLR9* SNPs on the overall survival in AML cases was studied by the Kaplan–Meier curve. AML patients who had the *TLR2* rs5743708 GA genotype had better overall survival. A previous study reported that the homozygous variant genotype of *TLR2* rs5743708 polymorphism was significantly associated with the shortest overall survival in Egyptian AML cases [16]. In addition, we observed that *TLR4* and *TLR9* gene polymorphisms did not affect the overall survival in our AML patients. These findings are in agreement with those reported by a previous study [19]. Considering that no associations were noticed between the seven *TLR* SNPs and the hazard of death, we may consider/suppose that the poorer outcome was conferred by other causes.

Regarding the relationship between investigated *TLR* SNPs and somatic mutations, we observed a significant association between the variant genotype of the *TLR9* rs5743836 and *NPM1* mutation and also a trend towards statistical significance between the *TLR4* rs11536889 SNP and *FLT3* ITD mutations. To the best of our knowledge, the impact of the mentioned *TLR2*, *TLR4* and *TLR9* variants on Caucasian adult AML cases have not been studied simultaneously. Rybka et al. investigated *TLR4* (rs4986790, rs4986791) and *TLR9* (rs5743836, rs187084) susceptibility to AML in a smaller group of subjects (62 AML cases and 126 controls) [16]. To our knowledge, this is the largest and most comprehensive study that investigates the relation of seven SNPs of the *TLR2*, *TLR4,* and *TLR9* genes with somatic mutation (*FLT3*, *NPM1*, *DNMT3A*), patient’s gender, age group, and characteristics of AML cases.

The present study has some limitations: a lack of data regarding *TLR2*, *TLR4,* and *TLR9* gene expression, and *CEBPA*, *IDH1*, *IDH2,* etc., mutation status was not investigated in all cases and heterogeneity of the AML patients included in the study (different AML subtype). Another limitation of this study is that this is an observational study of the association of *TLR2, TLR4* and *TLR9* polymorphisms, and *TLR9* haplotypes with AML risk and the overall survival of AML patients based on a single time-point evaluation. Taking into account the suppression of the immune system in active cancers such as AML, further prospective longitudinal studies with multiple time-point measures (e.g., before and after treatment) should be conducted in the future to establish if the TLRs of concern are being suppressed by the active cancer.

Moreover, further research that estimates the effect of gene–gene and gene–environment interactions may eventually provide a better and more comprehensive understanding.

## 5. Conclusions

Our findings suggest a potential association between the *TLR4* rs4986791 SNP and AML susceptibility. The combined variant genotypes of *TLR4* rs4986790 and *TLR4* rs4986791 appear to increase AML risk. In addition, the current findings indicate that the identification of patterns of genetic variations, in the form of the C-G-A haplotype of *TLR9* rather than the *TLR9* single nucleotide polymorphism, may represent a promising approach to predict the risk for developing acute myeloid leukemia.

## Figures and Tables

**Table 1:** Association analysis between variant genotypes/alleles of investigated *TLR* SNPs and odds of AML.

SNPs	AML Cases (n_1_ = 511)	Controls (n_2_ = 503)	Unadjusted		Adjusted ^(a)^	
			OR [95% CI]	*p*-Value	OR [95% CI]	*p*-Value
*TLR2* rs5743708						
GG	483 (94.52)	477 (94.83)	1.00 [Reference]		1.00 [Reference]	
GA	28 (5.48)	26 (5.17)	1.06 [0.61, 1.84]	0.826	1.03 [0.60, 1.79]	0.908
AA	0 (0.00)	0 (0.00)	NA		NA	
G allele	994 (97.26)	980 (97.42)	1.00 [Reference]		1.00 [Reference]	
A allele	28 (2.74)	26 (2.58)	1.06 [0.61, 1.84]	0.826	1.03 [0.60, 1.79]	0.908
HWE (*p*-value)	1.00	1.00				
*TLR4* rs11536889						
GG	367 (71.82)	356 (70.78)	1.00 [Reference]	0.922	1.00	1.00 [Reference]
GC	133 (26.03)	135 (26.84)	0.96 [0.72, 1.26]		0.92 [0.70, 1.23]	
CC	11 (2.15)	12 (2.39)	0.89 [0.39, 2.04]		0.89 [0.39, 2.06]	
GC + CC	144 (28.18)	147 (29.23)	0.95 [0.72, 1.25]	0.713	0.92 [0.70, 1.21]	0.561
G allele	867 (84.83)	847 (84.19)	1.00 [Reference]		1.00 [Reference]	
C allele	155 (15.17)	159 (15.81)	0.95 [0.75, 1.21]	0.691	0.93 [0.73, 1.19]	0.563
HWE (*p*-value)	1.00	1.00				
*TLR4* rs4986790						
AA	470 (91.98)	473 (94.04)	1.00 [Reference]	0.241	1.00 [Reference]	0.183
AG	40 (7.83)	30 (5.96)	1.34 [0.82, 2.19]		1.40 [0.86, 2.29]	
GG	1 (0.20)	0 (0.00)	NA		NA	
AG + GG	41 (8.03)	30 (5.96)	1.38 [0.84, 2.24]	0.198	1.44 [0.88, 2.35]	0.144
A allele	980 (95.89)	976 (97.02)	1.00 [Reference]		1.00 [Reference]	
G allele	42 (4.11)	30 (2.98)	1.39 [0.87, 2.25]	0.241	1.47 [0.91, 2.37]	0.118
HWE (*p*-value)	0.585	1.00				
*TLR4* rs4986791						
CC	464 (90.80)	472 (93.84)	1.00 [Reference]		1.00 [Reference]	
CT	47 (9.20)	30 (5.96)	1.59 [0.99, 2.56]	0.058	1.66 [1,00, 2.68]	0.059
TT	0 (0.00)	1 (0.20)	NA		NA	
CT + TT	47 (9.20)	31 (6.16)	1.54 [0.96, 2.47]	0.069	1.61 [1.001, 2.59] *	0.045 *
C allele	975 (95.40)	974 (96.82)	1.00 [Reference]		1.00 [Reference]	
T allele	47 (4.60)	32 (3.18)	1.48 [0.93, 2.34]	0.058	1.55 [0.97, 2.46]	0.064
HWE (*p*-value)	0.616	0.399				
*TLR9* rs352140						
CC	100 (19.57)	87 (17.30)	1.00 [Reference]	0.375	1.00 [Reference]	0.397
CT	242 (47.36)	230 (45.73)	0.92 [0.65, 1.29]		0.90 [0.64, 1.26]	
TT	169 (33.07)	186 (36.98)	0.79 [0.55, 1.13]		0.79 [0.55, 1.13]	
CT + TT	411 (80.43)	416 (82.71)	0.86 [0.63, 1.18]	0.351	0.85 [0.62, 1.17]	0.311
C allele	442 (43.25)	404 (40.16)	1.00 [Reference]		1.00 [Reference]	
T allele	580 (58.75)	602 (59.84)	0.89 [0.74, 1.05]	0.167	0.89 [0.75, 1.05]	0.175
HWE (*p*-value)	0.419	0.267				
*TLR9* rs187084						
AA	162 (31.70)	145 (28.83)	1.00 [Reference]	0.399	1.00 [Reference]	0.449
AG	248 (48.53)	243 (48.31)	0.91 [0.69, 1.22]		0.91 [0.69, 1.22]	
GG	101 (19.77)	115 (22.86)	0.79 [0.55, 1.11]		0.80 [0.56, 1.13]	
AG + GG	349 (68.30)	358 (71.17)	0.87 [0.67, 1.14]	0.319	0.88 [0.67, 1.15]	0.337
A allele	572 (55.97)	533 (52.98)				
G allele	450 (44.03)	473 (47.02)	1.00 [Reference]	0.182	1.00 [Reference]	0.210
HWE (*p*-value)	0.720	0.531	0.89 [0.75, 1.06]		0.90 [0.75, 1.06]	
*TLR9* rs5743836						
AA	370 (72.41)	368 (73.16)	1.00 [Reference]	0.767	1.00 [Reference]	0.751
AG	125 (24.46)	123 (24.45)	1.01 [0.76, 1.35]		1.01 [0.76, 1.35]	
GG	16 (3.13)	12 (2.39)	1.33 [0.62, 2.84]		1.34 [0.62, 2.88]	
AG + GG	141 (27.59)	135 (26.81)	1.04 [0.79, 1.37]	0.787	1.04 [0.79, 1.38]	0.763
A allele	865 (84.64)	859 (85.39)	1.00 [Reference]		1.00 [Reference]	
G allele	157 (15.36)	147 (14.61)	1.06 [0.83, 1.34]	0.643	1.06 [0.84, 1.35]	0.618
HWE (*p*-value)	0.175	0.595				

NA = not available; HWE: Hardy–Weinberg Equilibrium. [95% CI] = [lower limit, upper limit] of 95% Confidence Interval. ^(a)^ Adjusted for Age group (≥60 years versus <60 years) and sex (males versus female), * significant results at the α = 0.05 level.

**Table 2:** Linkage disequilibrium analysis for *TLR4* SNPs in AML patients and controls.

		*TLR4* rs11536889	*TLR4* rs4986790	*TLR4* rs4986791
*TLR4* rs11536889	D		−0.006	−0.007
	D’		0.987	0.993
	r		−0.086	−0.092
	χ^2^		7.63	8.69
	*p*-value		0.0057 *	0.0032 *
*TLR4* rs4986790	D	−0.005		0.034
	D’	0.989		0.874
	r	−0.075		0.824
	χ^2^	5.678		693.69
	*p*-value	0.0172 *		<0.001 *
*TLR4* rs4986791	D	−0.005	0.027	
	D’	0.988	0.931	
	r	−0.078	0.901	
	χ^2^	6.06	816.08	
	*p*-value	0.0138 *	<0.001 *	

D: Linkage disequilibrium estimate for each pair of SNPs; D’: scaled D having the values in the interval [−1, 1]; r: Correlation coefficient between any pair of SNPs; χ^2^: Chi-square statistic for linkage equilibrium (D = D’ = 0); *p*-value: Chi-square *p*-value; * significant results at the α = 0.05 level; the estimates above the main diagonal (marked in grey) were determined to AML group while the estimates below the main diagonal were determined in the control group.

**Table 3:** Linkage disequilibrium analysis for *TLR9* SNPs in AML patients and controls.

		*TLR9* rs352140	*TLR9* rs5743836	*TLR9* rs187084
*TLR9* rs352140	D		−0.051	−0.147
	D’		0.770	0.771
	r		−0.286	−0.597
	χ^2^		83.78	363.93
	*p*-value		< 0.001 *	< 0.001 *
*TLR9* rs5743836	D	−0.049		−0.051
	D’	0.829		0.751
	r	−0.284		−0.284
	χ^2^	79.40		82.33
	*p*-value	<0.001 *		<0.001 *
*TLR9* rs187084	D	−0.157	−0.059	
	D’	0.8317	0.857	
	r	−0.642	−0.334	
	χ^2^	414.39	112.18	
	*p*-value	<0.001 *	<0.001 *	

D: Linkage disequilibrium estimate for each pair of SNPs; D’: scaled D having the values in the interval [−1, 1]; r: Correlation coefficient between any pair of SNPs; χ^2^: Chi-square statistic for linkage equilibrium (D = D’ = 0); *p*-value: Chi-square *p*-value; * significant results at the α = 0.05 level; the estimates above the main diagonal (marked in grey) were determined to AML group while the estimates below the main diagonal were determined in the control group.

**Table 4:** Haplotype association analysis between *TLR 4* and *TLR9* SNPs with AML risk.

Gene	SNP1	SNP2	SNP3	Haplotype Relative Frequencies			Unadjusted OR ^(a)^ [95% CI]	Adjusted OR ^(b)^ [95% CI]	Global Score Statistics for the Adjusted Model
				All Sample	Control	AML Group			
*TLR9*	rs352140	rs5743836	rs187084						
*1*	T	A	G	0.409	0.437	0.382	1.00 [Reference]	1.00 [Reference]	Global-stat = 23.09, df = 7, *p*-value = 0.0017 *
*2*	C	A	A	0.374	0.370	0.377	1,17 [0.96, 1.42]	1.16 [0.96, 1,42]	
*3*	C	A	G	0.031	0.022	0.039	1.82 [1.06, 3.09] *	1.87 [1.10, 3.19] *	
*4*	C	G	A	0.006	0.002	0.012	5.75 [1.21, 9.98] *	5.54 [1.16, 26.51]*	
*5*	C	G	G	0.004	0.005	0.003	0.78 [0.19, 3.16]	0.81 [0.20, 3.27]	
*6*	T	A	A	0.035	0.023	0.047	2.01 [1.24, 3.28] *	2.01 [1.24, 3.28] *	
*7*	T	G	A	0.128	0.133	0.123	1.04 [0.78, 1.39]	1.04 [0.78, 1.38]	
*8*	T	G	G	0.009	0.004	0.014	3.19 [1.02, 9.98] *	3.31 [1.06, 10.34] *	
*TLR4*	rs4986790	rs4986891	rs11536889						
*1*	A	C	G	0.802	0.808	0.797	1.00 [Reference]	1.00 [Reference]	Global-stat = 5.64, df = 4, *p*-value = 0.2277
*2*	A	C	C	0.154	0.158	0.151	0.98 [0.77, 1.25]	0.96 [0.75, 1.23]	
*3*	A	T	G	0.006	0.003	0.009	2.49 [0.77, 7.99]	2.62 [0.81, 8.46]	
*4*	G	C	G	0.003	0.002	0.004	2.10 [0.47, 9.46]	2.27 [0.50, 10.26]	
*5*	G	T	G	0.032	0.027	0.036	1.32 [0.80, 2.20]	1.36 [0.82, 2.27]	
*6*	G	T	C	0.000	0.000	0.000	NA	NA	

Note. Haplotypes estimated from the three variants; NA = not available because of their low relative frequency (<0.002); ^(a)^ OR = odds ratio estimated from haplotype-based GLM regression without covariates; ^(b)^ adjusted for age group (≥60 years versus <60 years); statistical significant results: *p*-values smaller than 0.05 and the corresponding OR and 95% CI (Confidence interval) values are highlighted with *

**Table 5:** Frequency distribution of genotypic pairwise combinations of *TLR2*, *TLR4*, and *TLR9* SNPs and their association with AML risk.

Genotype	AML Cases (n_1_ = 511)	Controls (n_2_ = 503)	Adjusted ^(a)^	
			OR [95% CI]	*p*-Value
*TLR4* rs11536889 and *TLR4* rs4986790				
0	332	331	1.00 [Reference]	0.962
1	173	167	1.02 [0.78, 1.33]	
2	6	5	1.16 [0.35, 3.85]	
3	179	173	1.02 [0.79, 1.33]	0.857
*TLR4* rs11536889 and *TLR4* rs4986791				
0	327	330	1.00 [Reference]	0.820
1	177	168	1.05 [0.81, 1.37]	
2	7	5	1.37 [0.43, 4.36]	
3	184	173	1.06 [0.82, 1.37]	0.654
*TLR4* rs11536889 and *TLR9* rs352140				
0	67	57	1.00 [Reference]	0.505
1	333	329	0.84 [0.57, 1.24]	
2	111	117	0.77 [0.49, 1.20]	
3	444	446	0.82 [0.56, 1.20]	0.315
*TLR4* rs11536889 and *TLR2* rs5743708				
0	349	336	1.00 [Reference]	0.498
1	152	161	0.89 [0.61,1.16]	
2	10	6	1.45 [0.52, 4.06]	
3	162	167	0.91 [0.70, 1.18]	0.474
*TLR4* rs11536889 and *TLR9* rs5743836				
0	266	251	1.00 [Reference]	0.317
1	205	222	0.86 [0.67, 1.12]	
2	40	30	1.21 [0.73, 2.01]	
3	245	252	0.91 [0.71, 1.16]	0.436
*TLR4* rs11536889 and *TLR9* rs187084				
0	107	103	1.00 [Reference]	0.281
1	315	295	1.03 [0.75, 1.41]	
2	89	105	0.79 [0.54, 1.18]	
3	404	400	0.97 [0.71, 1.31]	0.839
*TLR4* rs4986790 and *TLR4* rs4986791				
0	460	470	1.00 [Reference]	0.032 *
1	14	5	3.14 [1.12, 8.84]	
2	37	28	1.41 [0.85, 2.34]	
3	51	33	1.66 [1.05, 2.64]	0.028 *
*TLR4* rs4986790 and *TLR9* rs352140				
0	91	81	1.00 [Reference]	0.355
1	388	398	0.86 [0.62, 1.20]	
2	32	24	1.22 [0.66, 2.24]	
3	420	422	0.88 [0.63, 1.23]	0.454
*TLR4* rs4986790 and *TLR2* rs5743708				
0	443	449	1.00 [Reference]	0.331
1	67	52	1.32 [0.89, 1.94]	
2	1	2	0.56 [0.05, 6.27]	
3	68	54	1.29 [0.88, 1.89]	0.191
*TLR4* rs4986790 and *TLR9* rs5743836				
0	340	343	1.00 [Reference]	0.281
1	160	155	1.06 [0.81, 1.38]	
2	11	5	2.29 [0.78, 6.68]	
3	171	160	1.10 [0.84, 1.43]	0.499
*TLR4* rs4986790 and *TLR9* rs187084				
0	148	138	1.00 [Reference]	0.648
1	336	342	0.92 [0.70, 1.21]	
2	27	23	1.16 [0.63, 2.13]	
3	363	365	0.93 [0.71, 1.25]	0.621
*TLR4* rs4986790 and *TLR9* rs352140				
0	92	80	1.00 [Reference]	0.069
1	380	399	0.82 [0.59, 1.15]	
2	39	24	1.45 [0.80, 2.62]	
3	419	423	0.86 [0.62, 1.19]	0.359
*TLR4* rs4986791 and *TLR2* rs5743708				
0	437	448	1.00 [Reference]	0.160
1	73	53	1.43 [0.98, 2.08]	
2	1	2	0.57 [0.05, 6.35]	
3	74	55	1.40 [0.96, 2.03]	0.079
*TLR4* rs4986791 and *TLR9* rs5743836				
0	333	342	1.00 [Reference]	0.284
1	168	156	1.12 [0.86, 1.47]	
2	10	5	2.13 [0.72, 6.33]	
3	178	161	1.15 [0.89, 1.59]	0.287
*TLR4* rs4986791 and *TLR9* rs187084				
0	148	137	1.00 [Reference]	0.224
1	330	343	0.89 [0.67, 1.18]	
2	33	23	1.41 [0.79, 1.54]	
3	363	366	0,92 [0.70, 1.21]	0.567
*TLR9* rs352140 and *TLR2* rs5743708				
0	95	83	1.00 [Reference]	0.609
1	393	398	0.85 [0.61, 1.18]	
2	23	22	0.88 [0.46, 1.70]	
3	416	420	0.85 [0.61, 1.18]	0.324
*TLR9* rs352140 and *TLR9* rs5743836				
0	94	82	1.00 [Reference]	0.579
1	282	291	0.84 [0.60, 1.18]	
2	135	130	0.90 [0.61, 1.32]	
3	417	420	0.86 [0.62, 1.19]	0.354
*TLR9* rs352140 and *TLR9* rs187084				
0	87	72	1.00 [Reference]	0.480
1	88	88	0.83 [0.54, 1.27]	
2	336	343	0.81 [0.57, 1.14]	
3	424	431	0.81 [0.58, 1.14]	0.229
*TLR2* rs5743708and *TLR9* rs5743836				
0	353	350	1.00 [Reference]	0.823
1	147	145	1.01 [0.76, 1.32]	
2	11	8	1.34 [0.53, 3.38]	
3	158	153	1.02 [0.78, 1.34]	0.869
*TLR2* rs5743708 and *TLR9* rs187084				
0	155	136	1.00 [Reference]	0.390
1	335	350	0.84 [0.64, 1.11]	
2	21	17	1.07 [0.54, 2.12]	
3	356	367	0.85 [0.65, 1.12]	0.246
*TLR9* rs5743836 and *TLR9* rs187084				
0	104	83	1.00 [Reference]	0.156
1	324	347	0.75 [0.54, 1.04]	
2	83	73	0.92 [0.60, 1.41]	
3	407	420	0.78 [0.56, 1.07]	0.122

Note. Variant genotypes for each SNP: GA genotype for TLR2 rs5743708; GC + CC genotype for TLR4 rs11536889; AG + GG genotype for TLR4 rs4986790; CT + TT genotype for TLR4 rs4986791; CT + TT genotype for TLR9 rs352140; AG + GG genotype for TLR9 rs187084; AG + GG genotype for TLR9 rs5743836. Wild genotypes (WT) for each SNP: GG genotype for TLR2 rs5743708; TLR4 rs11536889; AA genotype for TLR4 rs4986790; CC genotype for TLR4 rs4986791, TLR9 rs352140; AA genotype for TLR9 rs187084 and TLR9 rs5743836. 0: double-WT genotype, 1: Heterozygous genotype (WT SNP1 + variant genotype SNP2 or variant genotype SNP1 + WT SNP2), 2: double variant genotype (variant genotype SNP1 + variant genotype SNP2), 3: Heterozygous or double variant genotype; ^(a)^ OR = odds ratio adjusted for Age group (≥60 years versus <60 years) and sex (males versus female); [95% CI] = [lower limit, upper limit] of 95% Confidence Interval; * significant results at the α = 0.05 level.

**Table 6:** Associations between different genotypes of studied SNPs and AML patients’ overall survival.

SNPs	Genotypes	AML patients (n = 511)	Deaths (n = 397)	MeST ^(a)^ (95% CI)	Log-Rank *p*-Value	Unadjusted HR [95% CI]	*p*-Value	Adjusted HR ^(b)^ [95% CI]	*p*-Value ^(b)^
*TLR2* rs5743708	GG	483	380	6 [5,7]		1.00 (Reference)		1.00 (Reference)	
	GA	28	17	12 [8,36]	0.012 *	0.54 [0.33, 0.88]	0.0137 *	0.73 [0.44, 1.26]	0.272
*TLR4* rs11536889	GG	367	281	6 [5,7]	0.905	1.00 (Reference)		1.00 (Reference)	
	GC + CC	144	116	7 [5,9]	0.700	1.04 [0.84, 1.29]	0.714	1.16 [0.91, 1.48]	0.225
*TLR4* rs4986790	AA	470	361	7 [5,8]	0.197	1.00 (Reference)		1.00 (Reference)	
	AG + GG	41	36	4 [2,7]	0.092	1.35 [0.96, 1.90]	0.089	1.37 [0.95, 1.98]	0.095
*TLR4* rs4986791	CC	464	367	7 [5,8]	0.070	1.00 (Reference)		1.00 (Reference)	
	CT	47	41	5 [3,7]		1.35 [0.98, 1.88]	0.069	1.28 [0.90, 1.81]	0.171
*TLR9* rs352140	CC	100	80	5 [4,8]	0.270	1.00 (Reference)		1.00 (Reference)	
	CT + TT	411	317	7 [5,8]	0.270	0.87 [0.68, 1.12]	0.275	0.90 [0.68, 1.18]	0.430
*TLR9* rs187084	AA	162	130	6 [5,8]	0.607	1.00 (Reference)		1.00 (Reference)	
	AG + GG	349	267	6 [5,8]	0.626	0.95 [0.77, 1.17]	0.641	0.94 [0.74, 1.18]	0.579
*TLR9* rs5743836	AA	370	285	6 [5,7]	0.836	1.00 (Reference)		1.00 (Reference)	
	AG + GG	141	112	7 [4,9]	0.703	0.96 [0.77, 1.19]	0.713	0.96 [0.75, 1.22]	0.727
Number of variant genotypes	0	49	38	7 [4,10]	0.558	1.00 (Reference)		1.00 (Reference)	
	1	41	33	5 [3,9]		1.24 [0.78, 1.99]	0.363	1.18 [0.69, 2.03]	0.542
	2	144	109	7 [5,8]		0.95 [0.65, 1.37]	0.772	0.94 [0.62, 1.43]	0.776
	3	143	105	7 [4,9]		0.96 [0.66, 1.39]	0.831	0.94 [0.61, 1.46]	0.786
	4	106	88	6 [4,9]		1.05 [0.72, 1.54]	0.799	1.05 [0.68, 1.61]	0.836
	5–7	28	24	5.5 [2,8]		1.63 [0.94, 2.83]	0.283	1.55 [0.88, 2.73]	0.317

^(a)^ MeST = median overall survival time (months); the variant genotypes used for the calculation were *TLR2* rs5743708 GA + *TLR4* rs11536889 GC/CC, *TLR4* rs4986790 AG/GG + *TLR4* rs4986791CT + *TLR9* rs352140 CT/TT + *TLR9* rs187084 AG/GG + *TLR9* rs5743836AG/GG; because there were 6 patients with 5 variant genotypes and one patient with 6 and 7 variant genotypes, respectively, we merged the 5, 6 and 7 variant genotypes into one category. ^(b)^ HR = Hazard Ratio adjusted for age group (≥60 years vs. <60 years); sex (male vs. female); type of AML (WHO classification); ELN 2017 risk groups; ECOG, Eastern Cooperative Oncologic Group Scale; chemotherapy dose (high dose, low dose); LDH, lactate dehydrogenase (≥600 IU/L vs. 600IU/L); PLT, platelet (>40,000 vs. ≤40,000 cells/mm^3^); WBC, white blood cell (≥50,000 cells/mm^3^ vs. <50,000 cells/mm^3^); hemoglobin level (<10 g/dL vs. ≥10 g/dL); hematocrit level (<36% vs. ≥36%); blasts (in bone marrow, ≥70% vs. <70%); somatic mutations (*FLT3*, *NPM1*, *DNMT3A*); * significant results at the α = 0.05 level.

## Data Availability

The data included in the present work are available upon appropriate request by contact with the corresponding author.

## Supplementary Materials

The following supporting information can be downloaded at: https://www.mdpi.com/article/10.3390/jpm12030409/s1, Table S1. Association between studied TLR SNPs and somatic mutations (FLT3, NPM1, DNMT3A).
