# Supplementary material for: Association of TLR4 Rs4986791 Polymorphism and TLR9 Haplotypes with Acute Myeloid Leukemia Susceptibility: A Case-Control Study of Adult Patients
Source: J Pers Med. 2022 Mar 6;12(3):409. doi: 10.3390/jpm12030409 (PMC8950293; doi:10.3390/jpm12030409)
Supplement: Supplementary file 1 [file jpm-12-00409-s001.zip › jpm-1592228-supplementary.pdf]

Table S1. Association between studied *TLR* SNPs and somatic mutations (*FLT3, NPM1, DNMT3A*)

**SNPs**	**Genotypes**	**FLT3_IDT⁻ n (%)**	**FLT3_IDT⁺ n (%)**	**p-value**	**NPM1⁻ n (%)**	**NPM1⁺ n (%)**	**p-value**	**DNMT3A⁻ n (%)**	**DNMT3A⁺ n (%)**	**p-value**
TLR2 rs5743708	Wild type	404 (94.2)	78 (96.3)	0.598	395 (94.0)	87 (96.7)	0.447	434 (94.3)	49 (96.1)	1.000
	Variant	25 (5.8)	3 (3.7)		25 (6.0)	3 (3.3)		26 (5.7)	2 (3.9)	
TLR4 rs11536889	Wild type	315 (73.4)	51 (63.0)	0.055	300 (71.4)	66 (73.3)	0.716	334 (72.6)	33 (64.7)	0.234
	Variant	114 (26.6)	30 (37.0)		120 (28.6)	24 (26.7)		126 (27.4)	18 (35.3)	
TLR4 rs4986790	Wild type	393 (91.6)	76 (93.8)	0.501	386 (91.9)	83 (92.2)	0.920	424 (92.2)	46 (90.2)	0.588
	Variant	36 (8.4)	5 (6.2)		34 (8.1)	7 (7.8)		36 (7.8)	5 (9.8)	
TLR4 rs4986791	Wild type	387 (90.2)	76 (93.8)	0.302	383 (91.2)	80 (88.9)	0.493	418 (90.9)	46 (90.2)	0.800
	Variant	42 (9.8)	5 (6.2)		37 (8.8)	10 (11.1)		42 (9.1)	5 (9.8)	
TLR9 rs352140	Wild type	87 (20.3)	13 (16.0)	0.379	78 (18.6)	22 (24.4)	0.203	87 (18.9)	13 (25.5)	0.261
	Variant	342 (79.7)	68 (84.0)		342 (81.4)	68 (75.6)		373 (81.1)	38 (74.5)	
TLR9 rs187084	Wild type	141 (32.9)	21 (25.9)	0.218	136 (32.4)	26 (28.9)	0.518	144 (31.3)	18 (35.3)	0.561
	Variant	288 (67.1)	60 (74.1)		284 (67.6)	64 (71.1)		316 (68.7)	33 (64.7)	
TLR9 rs5743836	Wild type	306 (71.3)	63 (77.8)	0.234	292 (69.5)	77 (85.6)	**0.002**	330 (71.7)	40 (78.4)	0.310
	Variant	123(28.7)	18 (22.2)		128 (30.5)	13 (14.4)		130 (28.3)	11 (21.6)	

n= number of cases; Variant genotypes for each SNP: GA genotype for TLR2 rs5743708; GC+CC genotype for TLR4 rs11536889; AG+GG genotype for TLR4 rs4986790; CT+TT genotype for TLR4 rs4986791; CT+TT genotype for TLR9 rs352140; AG+GG genotype for TLR9 rs187084; AG+GG genotype for TLR9 rs5743836; Wild genotypes (WT) for each SNP: GG genotype for TLR2 rs5743708; TLR4 rs11536889; AA genotype for TLR4 rs4986790; CC genotype for TLR4 rs4986791, TLR9 rs352140; AA genotype for TLR9 rs187084 and TLR9 rs5743836; p-values were obtained using Chi-square or Fisher's exact tests; significant p-values (<0.05) were highlighted with bold font;
